# Hemolytic uremic syndrome and kidney transplantation in uncontrolled donation after circulatory death (DCD): A two-case report 

**DOI:** 10.5414/CNCS110434

**Published:** 2021-05-25

**Authors:** Leonardo Caroti, Giuseppe Cestone, Lorenzo Di Maria, Marco Allinovi, Vicenzo Li Marzi, Sergio Serni, Calogero Lino Cirami

**Affiliations:** 1 Nephrology Unit, Careggi University Hospital, and; 2 Department of Urologic Robotic Surgery and Renal Transplantation, University of Florence, Careggi Hospital, Florence, Italy

**Keywords:** thrombotic microangiopathy (TMA), uncontrolled donation after circulatory death (uDCD), ischemia/reperfusion injury (IRI), anti-C5 treatment

## Abstract

Background: Hemolytic uremic syndrome (HUS) is a rare disease characterized by microangiopathic hemolysis, thrombocytopenia, and renal involvement. Complement-mediated atypical HUS (aHUS) is a result of genetic defects in the alternative complement pathway components or regulators. The introduction of eculizumab has improved renal and overall survival of aHUS patients. Nowadays, given organ shortage, it is necessary to consider kidney transplantation (KT) even in protocols with a high risk of HUS recurrence, such as from donation after circulatory death (DCD) donors. Here, we describe two patients with HUS who underwent a KT from an uncontrolled DCD (uDCD). Case summary: The first patient, affected by aHUS due to a heterozygous deletion in CFHR3-CFHR1 and a novel heterozygous variant in CFHR5 gene, underwent a KT with eculizumab prophylaxis. The patient did not experience a post-transplant aHUS recurrence. The second patient, who experienced an HUS episode characterized by a hypertensive crisis and with no underlying mutations in complement system genes, underwent a KT without eculizumab prophylaxis. At day 5, anti-complement treatment commenced due to hematological signs of thrombotic microangiopathy (TMA). After the introduction of eculizumab, we observed a stabilization of kidney function and hematological remission. Conclusion: We present herein two different patients with HUS who both underwent successful KT from uDCD donation under the umbrella of eculizumab therapy. Taking into account the importance of increasing the number of organs available for transplantation, uDCD could represent an additional resource in this subset of HUS patients.

## Introduction 

**Figure 1:**
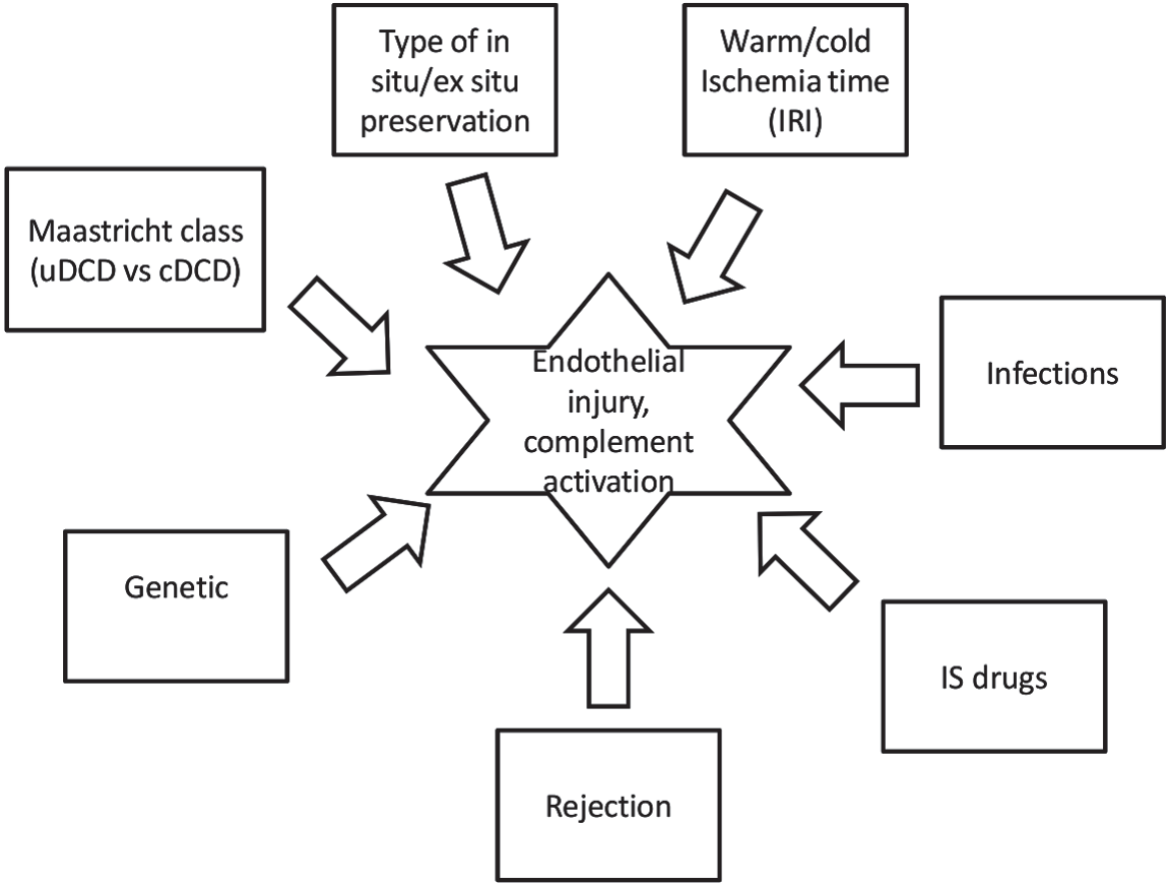
Risk factors for aHUS recurrence after uDCD. Modified from Zuber et al. [8]. uDCD = uncontrolled donation after circulatory death; cDCD = controlled donation after circulatory death; IS = immunosuppressive; IRI = ischemia-reperfusion injury.

Hemolytic uremic syndrome (HUS) is a rare disease characterized by microangiopathic hemolysis, thrombocytopenia, and renal involvement. In the recent literature, there are many reports about the classification of the disease that take into consideration the highly complex disease spectrum and the different etiologies [1]. Primary atypical HUS (aHUS) results from genetic defects in the alternative complement pathway components or regulators [2], resulting in an uncontrolled activation of this effector system, which leads to the formation of the terminal complement complex and consequent endothelial surface damage. Before the introduction of eculizumab, kidney transplantation (KT) had been a challenge for aHUS patients. In fact, between 50 and 80% of transplanted patients with aHUS showed a recurrence of the disease in the graft [3], and up to 80% of patients with a recurrence experienced transplanted organ failure [3, 4]. With the introduction of eculizumab, renal and overall survival from aHUS has dramatically improved, especially in patients who have had a KT; there was an inhibition of thrombotic microangiopathy (TMA), and most patients experienced a rapid improvement of renal function [5]. Over recent years, multiple retrospective cases describing the use of eculizumab to prevent recurrence after KT or de novo aHUS demonstrate a maintenance of allograft function, no additional post-transplant aHUS episodes, and no significant infectious complications [6, 7]. Genetic abnormalities are a very important aspect for clinical evaluation and for the prediction of the risk of recurrence. It is well-known that causative mutations in CFH, CFI, CFB, and C3 genes are associated with a high risk of aHUS recurrence after KT, while causative mutations in MCP gene are associated with a low risk of recurrence [3]. However, the prediction of aHUS recurrence depends on the complex interplay between genetic and environmental factors [8, 9]. Moreover, in patients with a low or moderate genetic risk of aHUS recurrence, a powerful trigger for complement activation could cause the disease to recur (Figure 1). Published data support the use of eculizumab prophylaxis when genetic complement analysis predicts a high-to-moderate risk of post-transplant aHUS recurrence [9, 10, 11]. In the pre-eculizumab era, KT strategies in aHUS patients were managed by trying to avoid possible triggers for endothelial injury; nowadays taking into account the organ shortage and the terminal complement C5 treatment availability, we should consider KT even in protocols with a high risk of recurrence, such as from donation after circulatory death (DCD). In secondary HUS caused by malignant hypertension, the implication of complement in the pathogenesis of the disease has been described, and some authors suggest anticomplement therapy in patients with organ damage [25]. Here, we describe two patients with HUS who underwent a KT from an uncontrolled DCD donor (Table 1). The medical records and data on genetic analysis of both patients with HUS who underwent a KT in our Nephrology Center were collected and reviewed using an electronic database. Data were retrieved from a single center study. HUS was diagnosed in patients who fulfilled the TMA criteria and after ruling out the following diseases: thrombotic thrombocytopenic purpura (TTP), typical HUS due to Shiga toxin-producing *Escherichia coli*, and secondary forms of TMA (such as malignancy, causative drugs, connective diseases, pregnancy). Genetic analysis for mutations in complement factors or regulators was performed on all patients. 

## Case histories 

### Case 1 

**Figure 2:**
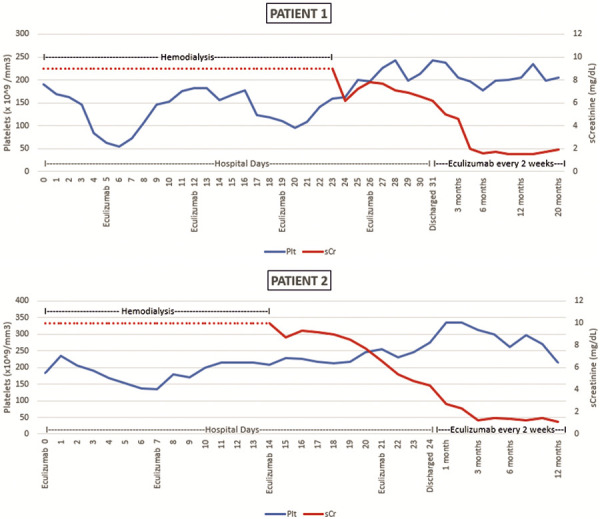
Evolution of platelet and serum creatinine after eculizumab initiation.

A 30-year-old Asian man developed TMA at the age of 24 years. He had gone to his local hospital with a 2-day history of progressive symptoms of dyspnea, abdominal pain, and vomiting. He denied previous pathologies or use of drugs. A physical examination showed that the patient was dyspnoic, pale, afebrile, and his blood pressure was 170/110 mmHg. Laboratory investigations showed profound anemia with hemoglobin of 6.4 g/dL, a low platelet count of 100 × 10^9^/mm^3^), signs of microangiopathic hemolysis with schistocytes on peripheral blood smear, low levels of haptoglobin, serum creatinine of 18.3 mg/dL, and serum urea of 264 mg/dL. Plasma exchange and hemodialysis were started immediately, and he received a packed red blood cell transfusion. All causes of TMA were investigated. Assessment of A disintegrin-like and metalloprotease with thrombospondin type 1 repeats (ADAMTS) 13 activity was normal (97%), ADAMTS 13 autoantibodies were negative, thereby ruling out TTP. Genetic analysis was performed, but no pathogenic mutations in complement genes were identified, the anti-CFH autoantibodies test was negative. When a complete normalization of hematological parameters was achieved, plasma exchange was stopped and a kidney biopsy performed. The histological examination showed TMA-like lesions with diffuse glomerulosclerosis, as seen in end-stage kidney disease. For this reason, terminal complement C5 treatment was not started, and hemodialysis was continued. At the age of 28, the patient received a cadaveric renal transplant from an uncontrolled DCD (uDCD). The patient was vaccinated against meningococcal infection before KT. Immunosuppressive therapy consisted of thymoglobulin, methylprednisolone, mycophenolate, and tacrolimus. No surgical complications occurred. A delayed graft function (DGF) was observed, and 5 days after transplantation the patient developed microangiopathic hemolytic anemia. Laboratory tests revealed hemoglobin of 7.6 g/dl, LDH 950 UI/L, platelets 50 × 10^9^/mm^3^, and peripheral blood smear revealed schistocytes. A kidney biopsy was not performed initially due to the clinical contraindication. Luminex test was negative, tacrolimus levels were 19.3 at day 3 and 9.3 at day 6. TMA recurrence was diagnosed, and eculizumab was started at the induction dose of 900 mg every week for 4 weeks and was then continued according to the standard schedule of 1,200 mg every 2 weeks. After the second administration of the drug, we observed a complete normalization of the patient’s hematological parameters. However, the resolution of TMA and improvement of renal function could be related to ischemia-reperfusion injury recovery, lowering of tacrolimus levels, and better control of blood pressure. The renal function started to improve from day 24 post transplant, and on day 31, the patient was discharged (Figure 2). A kidney biopsy was performed and showed no signs of rejection or TMA. 20 months after kidney transplantation, no new relapse of TMA has been observed, and kidney function is stable (sCr 1.9 mg/dL, eGFR 44 mL/min/1.73m^2^). Genetic analysis was performed again to exclude new pathogenic mutations in complement genes in order to discontinue eculizumab due the low risk of recurrence of the disease. 

### Case 2 

A 35-year-old Caucasian man with an unremarkable medical history was referred to his local hospital for severe headaches. At physical examination, the blood pressure was 190/100 mmHg, and a left ventricular hypertrophy was revealed on echocardiography. Laboratory tests showed no changes in kidney function, and the patient was discharged with antihypertensive therapy. At the age of 32, the patient went to the emergency department after a few days of nausea, vomiting, fatigue, and lethargy. Blood pressure was 240/130 mmHg. Laboratory tests showed serum creatinine 3.9 mg/dL and microangiopathic hemolytic anemia characterized by hemoglobin 9 g/dL, LDH 740 UI/L, schistocytes on peripheral blood smear, low levels of haptoglobin, and platelet count 125** × **10^9^/mm^3^. The patient was then transferred to our nephrology unit. Immunological tests (direct Coombs test, ANA, anti-DNA, ANCA) were negative, ADAMTS-13 was normal, while the C3 factor had decreased (0.4 g/L). Laboratory findings were compatible with the diagnosis of TMA, and consequently daily plasmapheresis was started. He underwent 9 sessions of plasmapheresis and intravenous pulses of methylprednisolone. Clinical improvement was observed after 3 weeks, with a complete hematological response. A kidney biopsy was performed showing the histopathological features of TMA. Genetic screening revealed a heterozygous CFHR3-CFHR1 deletion and a novel heterozygous variant c.1067G>A in CFHR5 gene. Eculizumab therapy was initiated, and after unsuccessfully discontinuing it after 1 year due to a relapse of TMA, treatment was continued biweekly, even after he started peritoneal dialysis at the age of 35, due to the high risk of hematological recurrence. At the age of 36**,** the patient received a cadaveric KT from an uDCD donor at our center. An additional prophylactic dose of eculizumab 900 mg was administered before surgery. The patient was vaccinated against meningococcal infection before KT. Immunosuppressive therapy consisted of thymoglobulin, methylprednisolone, mycophenolate mofetil, and delayed introduction of tacrolimus. No clinical or laboratory signs of TMA were detected. The patient developed a DGF and underwent hemodialysis sessions for the first 2 weeks. From the 15^th^ day post-transplantation we observed a progressive improvement in renal function. Serum creatinine was 4.2 mg/dL at discharge. After 12 months, serum creatinine was 1.13 mg/dL, eGFR 81 mL/min/1.73m^2^, with no recurrence of TMA (Figure 2). The treatment with eculizumab will continued due the risk of the recurrence of the disease. 

## Discussion 

In recent years, there has been a marked increase in the number of transplants from DCD donors [12, 13]. At our center, where the DCD program was started in 2016 [18], there have been almost exclusively uDCD donors (category IIa from the modified Maastricht classification). In Italy, a 20-minute period of observation is required after cardiorespiratory arrest before death can be attested to. This, as well as an increased incidence of primary non-function (PNF) and DGF in kidney recipients from uDCD donors [14, 15], may explain a condition of prolonged warm ischemia time. Moreover, kidneys from DCD donors are particularly vulnerable to the effects of cold ischemia time. It is well known that ischemia-reperfusion injury (IRI) is a crucial factor in the development of endothelial damage and complement activation, leading to inhibition of oxidative metabolism, depletion of ATP, increase in anaerobic glycolysis, inhibition of the Na/K ATPase pump, leucocyte recruitment in the graft, increased reactive oxygen species (ROS), and chemokines/chemotactic cytokines [16, 17]. Together, these factors explain the endothelial injury and dysfunction that can lead to complement overactivation, particularly in aHUS patients with a strong genetic susceptibility. However, use of eculizumab has not been shown to decrease the risk of DGF. In our donor service area, we adopted a DCD protocol of in situ preservation of abdominal organs with compartmentalized normothermic regional perfusion with extracorporeal membrane oxygenation (nECMO) and ex situ preservation of kidneys with hypothermic machine perfusion [18, 19]; normothermic regional perfusion (nRP) has been associated with a reduced risk for DGF if compared to in situ cooling [14]. The adopted immunosuppressive regimen provides a reduced tacrolimus concentration in order to avoid vascular renal injury. Therefore, an initial risk stratification for potential endothelial injury and complement activation based on different procedures, recipients’ genetic background, and donors’ clinical characteristics (in particular, age, comorbidities, duration of asystolic ischemia, type of in situ/ex situ preservation technique used) allows us to personalize the therapeutic protocol [20, 21]. In this report, the first patient was initially suspected to have a severe hypertension-related TMA, on the basis of medical history and echocardiogram. Considering these data and the negative genetic analysis for mutations in component or regulatory proteins of the complement cascade, we decided to perform a KT without eculizumab prophylaxis. However, eculizumab was started at day 5 post transplant due to subclinical TMA, that was potentially related to the ischemic damage induced by the KT from uDCD, in order to avoid potential further endothelial damage that could have influenced the outcome of the transplant in a probably non-complement-mediated TMA. This decision also took into account that a graft biopsy was not performed due to clinical contraindication. We observed a prompt hematological TMA remission and an improvement in kidney function, despite the delay in starting the treatment. It is still unclear if some secondary forms of HUS belong to the spectrum of complement-mediated aHUS [22]; indeed, a transient complement activation in secondary TMAs cannot be completely dismissed. Although much data suggest that secondary HUS and aHUS have no common genetic risk factors, a transient complement activation in secondary HUS cannot be discounted either. Moreover, the absence of genetic abnormalities does not exclude the role of complement in secondary HUS, and many case reports suggest a possible benefit of eculizumab in this setting. Complement overactivation, described even without complement genetic variants, may act as a “second hit”, which could perpetuate HUS and endothelial damage [23, 24, 25]. The second patient was affected by aHUS with a heterozygous deletion in CFHR3-CFHR1 deletion, considered a common benign variant that has been reported to be found in 3.3 – 6.7% of the Japanese population [26] and a novel heterozygous variant c.1067G>A in CFHR5 gene, of unknown significance. However, these genetic variants may represent a predisposition for the development of aHUS in the setting of KT from uDCD donors characterized by potential additional triggers, in particular if associated with other potential causative factors [27, 28]. The patient was in treatment with eculizumab at the time of the KT and experienced neither post-transplant aHUS recurrence nor evidence of progressing subclinical hematological TMA, in line with previously published literature. Current guidelines on eculizumab prophylaxis before KT in aHUS patients recommend a strategy targeting genetic abnormalities rather than the transplantation procedure [9]. Currently, to our knowledge, there are no data on KT from uDCD in patients with aHUS. Despite few recent data that supports a rescue approach with anti-complement therapy in patients with low risk of IRI [29], we recommend a protocol with eculizumab prophylaxis in all HUS patients who undergo a KT from uDCD. Subsequent checks to potentially discontinue eculizumab should depend on the genetic background. A rescue therapy with eculizumab in DCD could however be problematic for a number of reasons: an early TMA diagnosis is difficult, DGF is very common, as is an increase in the LDH and, in most of the cases, a kidney biopsy cannot be performed before the first week after a KT. Despite the limitations of a retrospective report of only two cases, this report supports the need to increase the number of organs available for transplantation from uDCD donors in patients with high risk of disease recurrence, such as those affected by HUS. 

## Conclusion 

Donation after circulatory death is one of the most important innovations in transplant proceedings over the last years and has increased the number of organs available for transplantation. Patients with HUS are characterized by a risk of post-transplant disease recurrence. Due to concerns regarding the additional warm ischemic damage in uDCD organs, uDCD donation in these patients could cause endothelial injury at transplant, and activate complement, triggering disease relapse. We have presented two patients who underwent a successful KT from uDCD under the umbrella of eculizumab regardless of pathogenetic mutations in complement genes. Given the importance of increasing the number of organs available for transplantation, especially for patients at high risk of complications in the pre-transplant course, uDCD could represent an additional resource in this subset of patients. 

## Informed consent statement 

Both patients gave informed consent for publication of scientific data. 

## Funding 

None. 

## Conflict of interest 

The authors have no conflict of interest to declare. 

**Table 1.** Patient/donor characteristics and renal outcomes.

Patient characteristics										
	Case 1					Case 2				
Age	30					36				
Sex	Male					Male				
Race	Asian					Caucasian				
Cause of ESRD	HUS					HUS				
Complement abnormality (Genetic test)	Negative					Heterozygous CFHR3-CFHR1 deletion and heterozygous variant c.1067G>A in CFHR5 gene				
Treatment	Hemodialysis					Peritoneal dialysis				
Eculizumab pre-tx	No					Yes				
DCD donor characteristics										
	Case 1					Case 2				
Age	40					46				
Total WIT (minutes)	155					157				
Cold ischemia time (h)	12					13				
Karpinsky score	2					4				
Type of in situ preservation	nRP					nRP				
Type of ex situ preservation	hMP					hMP				
Maastricht classification of DCD	IIa					IIa				
Transplant outcomes and characteristics										
	Case 1					Case 2				
DGF	24 days					14 days				
Immunosuppressive therapy	rATG + Tac + MMF + steroids					rATG + Tac + MMF + steroids				
Days of eculizumab doses	5, 12, 19, 26 (900 mg) Every 2 weeks (1,200 mg)					0, 7, 14, 21 (900 mg) Every 2 weeks (1,200 mg)				
N° of transplant	1					1				
Follow-up (months)	20					12				
Post-transplant relapse	Yes					No				
Renal allograft biopsy	Not performed					Not performed				
	1	4	12	24	54	1	4	12	24	54
	Weeks post-Tx					Weeks post-Tx				
Hemoglobin (g/dL)	8.5	9.1	13	11.6	13.8	8.6	10.5	10.7	13	14.2
Platelets (10^9^/mm^3^)	72	242	197	177	205	134	336	313	262	215
sCreatinine (mg/dL)	HD	6.2	2.08	1.6	1.58	HD	2,7	1.23	1.34	1.13
LDH	950	379	220	/	/	714	429	200	/	/

HUS = hemolytic uremic syndrome; WIT = warm-ischemia time; DCD = deceased cardiac donation; DGF = delayed graft function; nRP = normothermic regional perfusion; hMP = hypothermic machine perfusion; TAC = tacrolimus; MMF = mycophenolate mofetil.
